# Cancers du sein bilatéraux synchrones et grossesse a l'institut Joliot Curie de Dakar (Sénégal)

**DOI:** 10.11604/pamj.2016.23.142.8576

**Published:** 2016-03-30

**Authors:** Nayi Zongo, Yobi Alexis Sawadogo, Some Ollo Roland Some, Sidpawalmdé Carine Bagre, Sidy Ka, Doudou Diouf, Mamadou Moustapha Dieng, Papa Macoumba Gaye, Ahmadou Dem

**Affiliations:** 1 Département de Chirurgie, Service de Chirurgie Viscérale et Digestive, Centre Hospitalier Universitaire Yalgado Ouédraogo, Ouagadougou, Burkina Faso; 2 Service de Gynécologie Obstétrique, Centre Hospitalier Universitaire Yalgado Ouédraogo, Ouagadougou, Burkina Faso; 3 Département de chirurgie, Centre Hospitalier Universitaire Sanousourou, Bobo Dioulasso, Burkina Faso; 4 Institut de cancérologie Joliot Curie, Centre Hospitalier Universitaire Aristide le Dantec, Dakar, Sénégal; 5 Institut de Cancérologie Joliot Curie, Centre Hospitalier Universitaire Aristide Le Dantec, Dakar, Sénégal

**Keywords:** Cancers du sein bilatéraux, grossesse, pronostic, Breast, bilateral, cancer, pregnancy, prognosis

## Abstract

Décrire notre stratégie diagnostique et thérapeutique dans les cancers du sein bilatéraux synchrones pendant la grossesse. L’âge gestationnel au moment du diagnostic du cancer était respectivement de 7; 21 et 25 semaines. Il s'agissait de stade IV et IIIA respectivement dans deux et un cas. Elles ont toutes bénéficié d'une chimiothérapie dans deux cas pendant la grossesse (6TEC et 3AC) et dans un cas après l'accouchement. Une mastectomie bilatérale a été réalisée dans un cas. Une patiente est décédée. Les autres étaient vivantes mais toutes métastatiques. Une hypotrophie fœtale a été notée dans un cas. Le diagnostic du cancer est tardif dans cette association. La chirurgie est faisable et le type de chirurgie serait seulement fonction du stade du cancer. La chimiothérapie est possible avec moins de complications fœtales aux deux derniers trimestres.

## Abstract (en)

To describe our diagnostic and therapeutic strategy against synchronous, bilateral breast cancer during the pregnancy. Gestational age at diagnosis of cancer was 7; 21 and 25 weeks respectively. Patients had stage IV and IIIA of breast cancer in two and one case respectively. They all received chemotherapy, two cases during pregnancy (6TEC and 3AC) and one case after delivery. Bilateral mastectomy was performed in one case. One patient died. The others were alive but all metastatic. Fetal growth restriction was noted in one case. This association leads to delayed diagnosis of cancer. Surgery is feasible and the type of intervention is only determined by the cancer stage. Chemotherapy is feasible and is associated with less foetal complications in the last two quarters of pregnancy.

## Introduction

Les cancers diagnostiqués pendant la grossesse ou dans l'année après l'accouchement sont considérés comme cancers du sein associés à la grossesse [1]. L'association cancer du sein et grossesse n'est pas un événement rare à cause de l'augmentation de l'incidence de ce cancer chez les femmes jeunes. Cependant l'association cancer du sein bilatéral et grossesse reste exceptionnelle. Nous rapportons 3 cas dans le but de décrire notre stratégie diagnostique et thérapeutique.

## Patient et observation

### Méthodes

Notre étude s'est déroulée à l'institut Joliot Curie de Dakar située au centre hospitalier universitaire Aristide Le Dantec. Elle a concerné toutes les patientes ayant consultés à l'institut Joliot Curie quel que soit leur provenance pour des tumeurs mammaires bilatérales. Seules les patientes porteuse d'une grossesse et d'un cancer du sein bilatérale histologiquement confirmé ont été retenues. Les stades diagnostiques, les modalités thérapeutiques et évolutives ont été pris en compte.

### Observations

**Figure 1:**
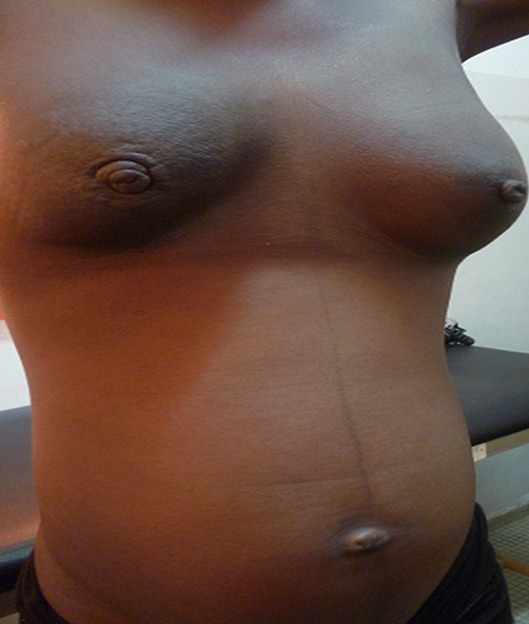
Cancer du sein bilatéral sur grossesse

**Cas 1:** MF, 22 ans, G1P0, étudiante, célibataire, a consulté le 23/12/10 à l'hôpital principal de Dakar pour des douleurs mammaires bilatérales. Le début remonterait à 5 mois par la survenue d'une masse inflammatoire du sein droit, puis deux mois plus tard du sein gauche. Elle a eu ses ménarches à 18 ans. Il n'y avait pas d'antécédents de pathologies mammaires dans sa famille. L'examen a mis en évidence un bon état général OMS 0, une mastite carcinomateuse bilatérale, douloureuse à la palpation, avec des adénopathies axillaires bilatérales fixées et sus-claviculaires. L'histologie a noté un adénocarcinome canalaire infiltrant bilatéral, moyennement différencié, SBR III, triple négatif (RH-, HER2 1+), avec un Ki à 80%. Le scanner thoraco-abdomino-pelvien a mis en évidence des localisations secondaires hépatiques, pulmonaires et vertébrales. L’échographie fœtale a mis en évidence une grossesse monofoetale, normo évolutive de 21 semaines. Les CA 15-3 étaient 10 fois la normale. Au total, il s'agissait d'un CCI, triple négatif, bilatéral, T4dN3cM1 à droite et T4dN2aM1 à gauche. Après 3 cures de taxotère-doxorubicine, on notait un bon état général, OMS 1, une réponse tumorale estimée à 70% au niveau mammaire, avec une diminution du phénomène inflammatoire, une réduction de la masse mammaire droite de 8 à 3 cm, et une masse gauche devenue infra centimétrique (Figure 1). Elle était dyspnéique en rapport avec une persistance de la pleurésie. Les drogues étaient relativement bien tolérées sans réaction allergique, avec néanmoins une alopécie grade 3, une mucite grade 1, des nausées/vomissements grade 2. Des ponctions itératives du liquide pleural furent réalisées améliorant la respiration. La cytologie du liquide pleural était positive. Un accouchement par césarienne programmée au-delà de 34 semaines d'aménorrhée après maturation pulmonaire a été réalisé. Elle a permis l'extraction par le pôle céphalique d'un nouveau-né de sexe masculin, pesant 2000g, avec un APGAR à 9. L’état respiratoire a été transitoirement amélioré après l'accouchement puis elle a été à nouveau dyspnéique avec nécessité de ponctions pleurales à répétition. Devant la progression rapide de la maladie, l'altération de l’état général, les problèmes financiers empêchant la patiente de débuter une seconde ligne métastatique de chimiothérapie, elle est partie dans son village à Fatick pour poursuivre ses soins. Réadmise d'urgence, elle est décédée le 6 février 2012 dans un tableau de détresse respiratoire et d'altération de l’état général. Le nourrisson se portait toujours bien.

**Cas 2:** FS, 25 ans, G2P1, porteuse d'une grossesse évolutive de 7 semaines d'aménorrhée, résidant à Thiès, a été adressée du CHR de Thiès à l'institut Joliot Curie de Dakar pour une tumeur mammaire bilatérale. Le début remonterait au mois de février 2011 par l'apparition d'un nodule du sein droit ayant nécessité une consultation au CHR de Thiès en avril 2011. L'examen clinique retrouvait un nodule du prolongement axillaire droit, indolore, mobile par rapport aux deux plans. L’échographie et la cytologie étaient en accord avec la clinique sur un trépied concordant bénin. Une nodulectomie a été faite. Son étude histologique retrouvait un adénocarcinome du QSE du sein droit SBR III, triple négatif. La patiente fut alors référée au centre du cancer pour une meilleure prise en charge. Dans ses antécédents, on notait un cancer mammaire chez la mère. Admise, le 9/11/2011 après son accouchement, on notait un bon état général, OMS 1, un score de Glasgow à 15. Les seins étaient symétriques et l'examen notait: une masse du prolongement axillaire droite en regard de la cicatrice de nodulectomie, fixée à la peau et mobile par rapport au plan profond. Elle mesurait 6 cm X 4 cm, avec des contours flous, légèrement sensible; une masse du prolongement axillaire du sein gauche, indolore, mobile par rapport au plan profond et fixée à la peau, mesurant 7 x 6 cm, donnant un aspect en miroir avec la masse du sein droit; des adénopathies axillaires bilatérales fixées et susclaviculaires droites. La mammographie notait au niveau du sein droit une grosse opacité de 5 cm du QSE avec des contours festonnés dans un tissu glandulaire dense ainsi qu'un épaississement de la peau en regard. Au niveau du sein gauche, elle retrouvait une grosse opacité de 7 cm du QSE à contours irréguliers. Il s'agissait donc de nodules bilatéraux BIRADS-ACR V. Le scanner thoraco-abdomino-pelvien a objectivé trois micronodules parenchymateux du lobe supérieur du poumon droit et des lésions hépatiques focales d'aspects métastatiques. Au total, il s'agissait d'un adénocarcinome canalaire infiltrant bilatéral du sein, moyennement différencié, SBR II, triple négatif, classé T4bN3cM1 à droite et T4bN2aM1 à gauche, soit un stade IV. Une première ligne métastatique à base de CMF a été instaurée. La tolérance a été bonne mais après 6 cures, il n'y pas eu de réponse notable. La patiente était en attente d'une seconde ligne métastatique.

**Figure 2:**
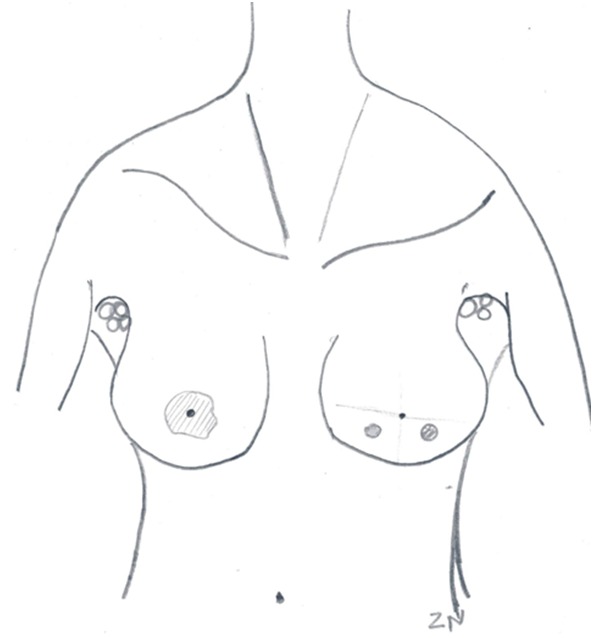
Masses mammaires bilatérales, multifocales à gauche

**Cas 3:** MAML, 33 ans, G4P4, trois enfants vivants, un décédé, résidant à Dakar, est adressée le 4 octobre 2011 de l'hôpital principal pour une tumeur mammaire bilatérale. Le début remonterait à 10 mois par la survenue d'un nodule au niveau du sein droit associé à une adénopathie axillaire droite. Trois mois plus tard est apparu un nodule au niveau du sein gauche. Elle a consulté à l'Hôpital Principal alors qu'elle était en grossesse de 23 semaines d'aménorrhées. Elle a eu ses ménarches à 16 ans. On ne notait pas d'antécédent de cancer mammaire ou gynécologique dans sa famille. La biopsie des masses mammaires ont mis en évidence au cours du sixième mois de gestité un carcinome mammaire bilatéral. L'option préconisée est la conservation de la grossesse jusqu′à 34 semaines d'aménorrhées tout en débutant une chimiothérapie néoadjuvante. Après 3 cures de AC60 et un accouchement par césarienne le 26/07/11 d'un enfant sain, elle est transférée au centre du cancer de Dakar, pour une meilleure prise en charge. A son admission, elle avait un bon état général OMS 0, une conscience claire. On notait au niveau du sein droit, la présence d'une tumeur retro-aréolaire, aux contours flous, d'environ 3,5 cm de diamètre, mobile par rapport aux deux plans, avec des adénopathies axillaires fixées homolatérales. Les quadrants inféro-externe et inféro-interne du sein gauche était chacun le siège d'un nodule mobile, centimétriques, avec des adénopathies axillaires ipsilatérales mobiles (Figure 2). L’échographie retrouvait des formations hypoéchogènes à contours bosselés, mesurant respectivement 38 mm, 8,3 mm et 7,5 mm et la présence d'adénopathies satellites axillaires bilatérales plus importantes à droite. L'histologie des pièces biopsiques mettait en évidence un carcinome canalaire infiltrant, SBR III, triple négatif. Au total, il s'agissait de carcinome canalaire infiltrant triple négatif, de grade III, sur grossesse, T2N2aM0 à droite et T1N1M0 à gauche. L'IRM thoraco-abdominale n'objectivait pas de lésions secondaires. Les CA15-3 étaient à 112 U/ml. Après 3 autres cures d'AC, la réponse était estimée à 100% à droite et 80% à gauche. Les effets de la chimiothérapie étaient représentés par une anémie grade 2 après la troisième cure ayant nécessité une transfusion de 2 concentrés globulaires avant la quatrième cure, une alopécie grade 3, une toxicité cutanée grade 2. La patiente a bénéficié d'une MCA bilatérale le 18 janvier 2012. Les suites opératoires ont été simples néanmoins marquées par une mobilisation douloureuse au début de l’épaule droite de façon transitoire. Elle présentait des adénopathies susclaviculaires droites et bénéficiait d'une chimiothérapie adjuvante à base de taxotère et de doxorubicine.

## Discussion

Les cancers du sein diagnostiqués pendant la grossesse ou dans l'année après l'accouchement sont considérés comme cancers du sein associés à la grossesse [1]. La survenue à un âge jeune des CSBS, avant la ménopause, explique la coexistence d'une grossesse. L'association cancer du sein unilatéral et grossesse reste un événement rare malgré l'augmentation de l'incidence de ce cancer chez les femmes jeunes et les grossesses devenues de plus en plus tardives [1, 2]. Son incidence est estimée entre 0,2% et 3,8%. Dix pour cent des femmes de moins de 40 ans ayant un cancer de sein sont enceintes lors du diagnostic [3]. L'association cancer du sein bilatéral et grossesse reste exceptionnelle. Seul un cas a été rapporté dans la littérature. Il s'agissait d'un lymphome de Burkitt bilatéral du sein survenu en postpartum [4]. Cette coexistence, bien qu'il ne semble pas y avoir de relation de causalité, pose des problèmes diagnostiques et thérapeutiques différents. Le diagnostic du cancer est tardif dans cette association. Les examens invasifs sont à éviter sauf s'ils modifient de façon importante la stratégie thérapeutique et le pronostic maternel. Pendant la grossesse, du fait de l'augmentation de volume, de l'engorgement, de l'hypervascularisation et de la prolifération du tissu mammaire, l'examen clinique est difficile et la mammographie moins sensible. L’échographie non irradiante, inoffensive pour la mère et le fœtus est l'examen de référence et guide la biopsie. Une radiographie thoracique et une échographie abdominale constituent les examens du bilan d'extension. Le scanner n'est pas recommandé [1, 2]. L'IRM avec injection de gadolinium, moins irradiante que le scanner est envisageable seulement quand elle risque de modifier la décision thérapeutique: en cas de suspicion de multifocalité avant de choisir entre chirurgie première ou chimiothérapie néoadjuvante, en cas de carcinome lobulaire [1]. L'interruption de grossesse n'améliore pas le pronostic. Sa poursuite serait même corrélée à une meilleure survie maternelle [1]. Cela est rassurant et une bonne nouvelle dans le contexte africain où une interruption de grossesse même thérapeutique serait mal comprise et mal vécue par la patiente. Aucune de nos trois patientes n'avait envisagé une interruption de grossesse. Le cas n^°^2 a même retardé sa consultation d'oncologie pour attendre son accouchement avant d'entreprendre tout traitement malgré les risques carcinologiques que cela impliquait. L'interruption de grossesse ne représente pas un acte thérapeutique y compris chez la femme métastatique. La seule exception serait la patiente métastatique HER2 positif pour laquelle il y a une indication de trastuzumab [2]. La chirurgie bien que souvent plus hémorragique est radicale ou conservatrice en fonction du stade du cancer. La chimiothérapie est possible avec moins de complications fœtales aux deux derniers trimestres [5]. En effet, après le premier trimestre où les antimétabolites et les alkylants sont les plus foetotoxiques, on ne constate pas de risque de malformations fœtales supérieur à celui de la population de femmes ne recevant pas de chimiothérapie [5]. Le trastuzumab dans les HER2 positifs n'a pas d'indication actuellement pendant la grossesse, car responsable d'oligo-anhydroamnios [6]. La radiothérapie et le tamoxifène sont contre-indiqués pendant la grossesse [7].

## Conclusion

L'association cancer du sein bilatéral et grossesse est exceptionnelle. Le traitement ne doit pas attendre le terme de la grossesse. Il doit être bénéfique pour la mère tout en restant le moins toxique possible pour le fœtus. Ces conditions sont toutefois difficiles pour les cancers diagnostiqués au premier trimestre. Le pronostic maternel est mauvais.
